# ACP1 Genetic Polymorphism and Coronary Artery Disease: Evidence of Effects on Clinical Parameters of Cardiac Function

**DOI:** 10.4021/cr277w

**Published:** 2013-07-11

**Authors:** Fulvia Gloria-Bottini, Maria Banci, Patrizia Saccucci, Paolo Nardi, Mattia Scognamiglio, Federica Papetti, Sara Adanti, Andrea Magrini, Antonio Pellegrino, Egidio Bottini, Luigi Chiariello

**Affiliations:** a Department of Biomedicine and Prevention, University of Rome Tor Vergata, School of Medicine, Rome, Italy; b Department of Cardiology, Valmontone Hospital, Rome, Italy; c Department of Cardiac Surgery, University of Rome Tor Vergata, School of Medicine, Rome, Italy

**Keywords:** ACP1, CAD, LVEF, Genetic polymorphisms, Phosphatases, Cardiovascular diseases

## Abstract

**Background:**

Kinases and phosphatases have an important role in the susceptibility and clinical variability of cardiac diseases. We have recently reported an association between a phosphoprotein phosphatase controlled by Acid Phosphatase locus 1 (ACP1), and Coronary artery disease (CAD) suggesting an effect on the susceptibility to this disease. In the present note we have investigated a possible role of ACP1 in the variability of clinical parameters of cardiac function.

**Methods:**

We have studied 345 subjects admitted to Valmontone Hospital for cardiovascular diseases: 202 subjects with CAD and 143 without CAD, 53 subjects admitted to Cardiac Surgery Division of Tor Vergata University were also considered.

**Results:**

In diabetic patients with CAD there is a significant negative association between Left ventricular ejection fraction (LVEF) and ACP1 S isoform concentration. Genotypes with high S isoform concentration show a lower value of LVEF as compared to genotypes with low S isoform concentration. We have also found a significant positive association between cNYHA class and ACP1 S isoform. After surgical intervention, in subjects with high S isoform concentration the decrease of LVEF is more marked as compared to subjects with low S isoform concentration. Overall these observations indicate that high S isoform activity has negative effects on cardiac function. The observation in patients undergoing cardiac surgery confirms the negative association between high S isoform activity and LVEF.

**Conclusions:**

The present study suggests that ACP1 influences both susceptibility to CAD and clinical manifestations of the disease.

## Introduction

An important role of kinases in cardiac function is well established [1-8]. Less investigated is the role of phosphatases that contribute to modulate the effect of kinases. Genetic variability of kinases and phosphatases could have an important role in the susceptibility and clinical variability of cardiac diseases. We have recently reported an association between Coronary Artery Disease (CAD) and Acid Phosphatase locus 1 (ACP1) suggesting a role of ACP1 in the susceptibility to CAD [9].

In the present note we have investigated a possible role of ACP1 in the variability of clinical parameters of cardiac function in subjects with CAD.

### The genetic polymorphism of ACP1

ACP1 controls the synthesis of cytosolic low molecular weight protein tyrosine phosphatase (cLMWPTP) a polymorphic enzyme showing strong quantitative variations of total enzymatic activity among genotypes. In Caucasian population there are 6 genotypes attributed to the presence of 3 codominant alleles, ACP1 *A, ACP1 *B and ACP1 *C, at an autosomal locus [10].

The enzyme is composed of two isoforms, F and S that have different molecular and catalytic properties [11, 12] and different concentrations among ACP_1_ genotypes (Table 1).

**Table 1:** F and S Isoform Concentrations in Relation to the ACP1 Genotype

Total quantity of F (µg/mL RBC)		Total quantity of S (µg/mL RBC)	
*B/*B	16.4	*C/*C	20.6
*A/*B	12.0	*A/*C	12.7
*B/*C	11.3	*B/*C	12.1
*A/*A	7.9	*B/*B	3.9
*A/*C	7.5	*A/*B	3.4
*C/*C	5.7	*A/*A	3.3

The total enzymatic activity measured with p-nitrophenylphosphate as a substrate is in the order *A/*A < *A/*B < (*B/*B, *A/*C) < *B/*C < *C/*C [10].

Two separate functions have been attributed to cLMWPTP: phosphoprotein tyrosine phosphatase and flavin mononucleotide phosphatase [12].

The cLMWPTP dephosphorylates a negative phosphorylation site in the ZAP-70 tyrosine kinase in T cells [13]. This event leads to increased activation of this kinase and enhanced signalling from T cell antigen receptor.

The enzyme is involved in the negative modulation of insulin signal transduction [14]. Stefani et al [15] have studied the dephosphorylation of tyrosine phosphorylated synthetic peptides by rat liver phosphotyrosine protein phosphatase isoenzymes named A and B (which correspond to the human ACP1 F and S isoforms) and have found differences in the main kinetic parameters of A and B isoenzymes for insulin receptor and B3P phosphorylated peptides.

PDGF regulates cell growth and division and plays a significant role in angiogenesis and in the growth of blood vessels [16]. Recent studies suggest that PDGF exerts a protective role on contractile function of cardiomyocytes and protects cardiomyocytes from apoptotic cell death [17]. cLMWPTP is able to dephosphorylate platelet-derived growth factor (PDGF) receptors decreasing its activity as growth factor and as protective factor of cardiomyocyte.

## Material and Methods

We studied 345 subjects admitted to Valmontone Hospital for cardiovascular diseases: 202 with CAD and 143 without CAD (Table 2, 3). A study on the relationship between ACP1 genotype and behaviour of LVEF after surgical intervention has been carried out in a sample of 53 subjects admitted to Cardiac Surgery Division of Tor Vergata University (Table 4).

**Table 2:** Clinical Data in Subjects With CAD From Valmontone Hospital

Parameter	% Proportion
Infarction	40.5%
Major coronary lesions	83.2%
Bypass	35.2%
Angioplastic	27.1%
Gender (Female %)	47.4%
Smoking habit	65.4 %
Diabetes	34.2%

	Mean	SD
Age (years)	66.7	± 11.6
Body mass index (kg/m^2^)	27.36	± 5.2

**Table 3:** Clinical Data in Subjects Admitted to Valmontone Hospital for Cardiovascular Diseases Without CAD

Parameter	% Proportion
Gender (Female %)	65.8%
Defect of the hearth valves	32.5%
Hypertension^a^	58.3%
Cardiac hypertrophy^b^	44.4%
Dilated heart^c^	16.6%
Cardiac Arrhythmia^d^	49.3%
Smoking habit	37.8 %
Diabetes	19.4%

	Mean	SD
Age (years)	53.92	± 16.25
Body mass index (kg/m^2^)	26.43	± 5.35

^a^medicated against hypertension/arterial tension ≥ 130/85 mmHg; ^b^patients with thickness walls ≥ 11 mm; ^c^diameter diastolic left ventricular ≥ 56 mm; ^d^ patients with atrial fibrillation, sinusale arrhythmia, atrial ventricular blocks.

**Table 4:** Clinical Data in Subjects Admitted to Cardiac Surgery Division of Tor Vergata University. Subjects With CAD

Parameter	% Proportion
Gender (Female %)	30.1%
Major coronary lesions	100%
Infarction	67.9%
Hypertension^a^	92.4%
Cardiac hypertrophy^b^	73.5%
Dilated Heart^c^	11.3%
Smoking habit	72.2%
Cardiac Arrhythmia^d^	13.2 %
Diabetes^e^	47.1%

	Mean	SD
Age (years)	64.5	± 20.5
Body mass index (kg/m^2^)	31.2	± 4.6

^a^medicated against hypertension/arterial tension ≥ 130/85 mmHg; ^b^patients with thickness walls ≥ 11mm; ^c^diameter diastolic left ventricular ≥ 56 mm; ^d^patients with atrial fibrillation, sinusale arrhythmia, atrial ventricular blocks; ^e^medicated with anti-diabetic drugs/glycaemia ≥ 110 mg/L.

Patients from Valmontone Hospital have been considered in a previous study on the effect of ACP1 on susceptibility to CAD [9].

Informed written consent was obtained from each patient. The study protocol conforms to the Ethical Guidelines of the 1975 declaration of Helsinki and the investigation was approved by the Ethical Committee of the Hospital.

LV volumes and Left Ventricular Ejection Fraction (LVEF) were calculated using the modified Simpson’s rule, by tracing the endocardial border in 2-chamber view and 4-chamber view. End diastole was defined as the frame in the cardiac cycle in which the cardiac dimension is largest and end systole was best defined as the time in the cardiac cycle in which the cardiac dimension is smallest [18]. All determinations were carried out by the same person.

The severity of heart failure was evaluated according to New York Heart Association (NYHA) Classification [19].

The ACP1 polymorphism was studied using the restriction fragment length polymorphism polymerase chain reaction method as previously described [9].

F and S isoforms concentrations were assigned to each genotype according to the data in Table 1.

Correlation analysis has been performed by Pearson method, differences between means have been evaluated by Student t. Tests of independence have been performed by Pearson Chi square statistics. The strength of association has been evaluated by eta (η) statistic. The square (η^2^) of eta gives the proportion of variance of one variable explained by the other variable. Factor analysis was performed by principal component method. All analyses were carried out using SPSS programs [20].

## Results

Tables 2, 3 and 4 show clinical data of the study samples.

Table 5 shows the correlation between ACP1 parameters and LVEF in patients with cardiovascular diseases. In diabetic patients with CAD there is a highly significant negative correlation between S isoform and LVEF. The correlation with F isoform is positive but not statistically significant resulting in a statistically significant positive correlation between LVEF and F/S ratio.

**Table 5:** Correlation Between ACP_1_ Parameters and LVEF in Patients With CAD

	All patients		CAD		Non CAD	
	r	P	r	P	r	P
Total activity	-0.150	0.005	-0.137	0.049	-0.088	0.310
S isoform	-0.147	0.006	-0.176	0.012	0.000	0.994
F isoform	-0.004	0.943	0.052	0.455	-0.094	0.280
F/S ratio	0.109	0.042	0.159	0.023	-0.057	0.513

			Patients with CAD		
	Diabetics			Non Diabetics	
	r	P		r	P
Total activity	-0.247	0.039		-0.022	0.805
S isoform	-0.338	0.004		-0.036	0.691
F isoform	0.123	0.312		0.016	0.860
F/S ratio	0.289	0.015		0.047	0.607

Table 6 shows the correlation between ACP1 parameters and cNHYA class in patients with cardiovascular diseases. In diabetic patients with CAD there is a highly significant positive correlation between S isoform and cNYHA class. The correlation with F isoform is negative resulting in a highly significant negative correlation with F/S ratio.

**Table 6:** Correlation Between ACP1 Parameters and cNYHA in Patients With Cardiovascular Diseases

	All patients			CAD			Non CAD		
	r	P		r	P		r		P
Total activity	0.169	0.001		0.219	0.001		0.037		0.642
S isoform	0.193	0.000		0.281	0.000		0.023		0.773
F isoform	-0.019	0.706		-0.074	0.275		0.017		0.833
F/S ratio	-0.152	0.002		-0.240	0.000		-0.016		0.839

	Patients with CAD								
	Diabetics					Non Diabetics			
	r		P			r		P	
Total activity	0.285		0.014			0.164		0.059	
S isoform	0.508		0.000			0.133		0.128	
F isoform	-0.253		0.030			0.038		0.667	
F/S ratio	-0.467		0.000			-0.081		0.357	

New York Heart Association (NYHA) Classification: Class I: patients with no limitation of activities; they suffer no symptoms from ordinary activities. Class II: patients with slight, mild limitation of activity; they are comfortable with rest or with mild exertion. Class III: patients with marked limitation of activity; they are comfortable only at rest. Class IV: patients who should be at complete rest, confined to bed or chair; any physical activity brings on discomfort and symptoms occur at rest.

The data in Tables 5 and 6 indicate a negative effect of S isoform on cardiac function in diabetic subjects with CAD. In Table 7 we have carried out an anlysis considering two classes of ACP1 genotypes: those with high S isoform (genotypes *C/*C, *A/*C and *B/*C) vs those with low S isoform concentration (genotypes *A/*A, *A/*B and *B/*B). The data show in CAD diabetics a highly significant difference of both LVEF and cNYHA between the two classes of ACP_1_ genotypes.

**Table 7:** Parameters of Cardiac Function in Relation to ACP_1_ Genotype

	CAD Diabetics LVEF				CAD Non Diabetics LVEF			
	mean	S.E.	N°	P	mean	S.E.	N°	P
ACP_1_ genotypes with low S isoform activity	48.06	1.204	53		50.79	0.894	112	
				0.005				0.599
ACP_1_ genotypes with high S isoform activity	40.06	3.009	16		49.65	1.631	21	

	cNYHA				cNYHA			
	mean	S.E.	N°	P	mean	S.E.	N°	P
ACP_1_ genotypes with low S isoform activity	1.63	0.105	53		1.37	0.103	112	
				0.001				0.091
ACP_1_ genotypes with high S isoform activity	2.93	0.316	16		1.81	0.245	21	

Parametric analysis, ACP_1_ genotype has been grouped into 2 classes: low S isoform concentration (*A/*A, *A/*B and *B/*B) and high S isoform concentration (*C/*C, *A/*C and *B/*C), patients with CAD.

We have also examined the proportion of patients with a very low LVEF (≤ 40%) and the proportion of patients with a cNYHA class ≥ 2 in subjects carrying the genotypes with low and respectively with high S isoform activity. Among CAD diabetics the proportion of those with LVEF ≤ 40% is much higher in patients carrying ACP_1_ genotypes with high isoform concentration than in patients carrying ACP1 genotypes with low S isoform concentration (P = 0.02). This difference is not observed in non diabetic subjects. Among CAD diabetics the proportion of those with a cNYHA class ≥ 2 is much higher in ACP1 genotypes with high S isoform concentration than in ACP1 genotypes with low S isoform concentration (P = 0.001). Such difference is not observed in non diabetics (data not shown).

We have considered the effect of sex, history of previous infarction and age on the relationship of low LVEF and cNYHA class with ACP1 genotypes in CAD diabetics. No significant effect of gender has been observed. The association of cNYHA class and low LVEF with ACP1 genotypes is more marked in subjects with previous history of infarction. A highly significant effect of age is observed on the relationship between ACP_1_ genotype and both cNYHA class (η^2^ = 0.035 in subjects aging less than 65 yrs vs η^2^ = 0.533 in subjects aging more than 65 yrs) and low LVEF (h^2^ = 0.005 in subjects aging less than 65 yrs and h^2^ = 0.130 in subjects aging more than 65 yrs).

A principal component analysis has been performed considering LVEF, cNYHA, S isoform, sex, history of previous infarction and age. Two components resulted statistically significant: the first component shows in diabetic patients but not in non diabetic patients a clear association of S isoform with LVEF and cNYHA. This relationship is influenced by age in agreement with the results obtained by the above mentioned analysis (data not shown).

**Figure 1:**
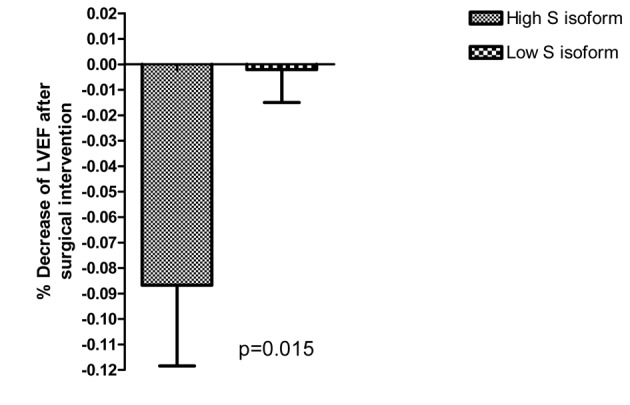
Behaviour of LVEF after surgical intervention in relation to S isoform activity.

In a sample of 53 patients we have considered the possible effect of ACP_1_ genotypes with high S isoform activity on the behaviour of LVEF after surgical intervention. Figure 1 shows that in subjects with high S isoform activity there is a significant decrease of LVEF while in other genotypes this decrease is minimal.

## Discussion

Our data suggest that high S isoform activity has negative effect on LVEF and cNYHA in diabetic subjects with coronary artery disease. The observation on the sample of patients undergoing cardiac surgery is in line with the negative association of high S isoform activity with LVEF.

The effects of ACP1 on LVEF seem to be specific for CAD since it is not present in patient admitted to the Hospital for other cardiac pathologies.

Although the exact biochemical mechanisms underlying cardiac function are not completely clear, the role of kinases and phosphatases are probably of prominent importance [8].

Influencing the phosphorylation status of insulin receptor and band 3 protein of cytoskeleton (B3P) ACP1 contributes to the regulation of glucose metabolism. Indeed low ACP1 activity is associated with increased glucose tolerance [21, 22]. On the other hand though the regulation of phosphorylation status of ZAP70 kinase shows influences on immunological functions [13]. Immunological mechanisms have been proposed for pathogenesis of atherosclerosis [23-25].

High S isoform activity may have negative effects on cardiac glucose metabolism resulting in negative effect on cardiac function. The marked negative association between S isoform activity and LVEF observed in diabetic subjects is in line with this interpretation.

Platelet Derived Growth Factor regulates cell growth and division and plays a significant role in angiogenesis and in the growth of blood vessels [16]. ACP1 is able to dephosphorylate PDGF receptors decreasing their activity as growth factor [26]. ACP1 genotypes with high S isoform activity may decrease PDGF activity with negative effect on contractile function of cardiomyocyte and on the protection from apoptotic cell death [17].
